# Self-Esteem and Resilience Differences Among Street Children Compared to Non-street Children in Limpopo Province of South Africa: A Baseline Study

**DOI:** 10.3389/fpubh.2021.542778

**Published:** 2021-04-23

**Authors:** Mokoena Patronella Maepa

**Affiliations:** Department of Clinical Psychology, School of Medicine, Sefako Makgatho Health Sciences University, Pretoria, South Africa

**Keywords:** South Africa, Limpopo Province, street children, non-street children, self-esteem, resilience

## Abstract

The phenomenon of street children is a challenging global social problem. Using an independent sample group design, this study explored the differences in self-esteem and resilience among street children and non-street children. A total of 300 (*N* = 300) street children with ages ranging from 8 to 18 years were selected using a purposive sampling method, while a total of 300 (*N* = 300) non-street children with ages ranging from 8 to 18 years were selected using a simple random sample to participate in this study. A questionnaire with three sections was used to collect data. Results of an independent sample *t*-test revealed that street children reported low self-esteem and poor resilience compared to non-street children. The study, therefore, concluded that street children and non-street children differ on self-esteem and resilience. It is recommended that social skills training be provided for the street children population.

## Introduction

Street children phenomenon is a global social problem. In South Africa, The Tshwane Alliance for Street Children (1) reported about 100,000 cases of street children. They are defined as persons who are below the age of 18 years who live on the streets on their own without any form of parental or adult care (National Coalition for the Homeless as cited in Ligon (2)). They are identified as children who are in difficult or challenging circumstances some associated with chronic poverty in their homes (3). They are predisposed to psychosocial problems such as substance use (4) and exposure to violence and aggression (5), being victims of social ills such as rape, violence, and different acts of aggression (6). Their challenges also include sexual and reproductive health such as HIV/AIDS and other sexually transmitted diseases (7). They are also prone to untimely deaths (8).

The challenges associated with street life exacerbate the behavioral issues of homelessness (9). They have to find ways of coping with the harsh living conditions on the street. Their survival methods include risky behavior (10) such as survival sex (9) and crime in the form of car/housebreaking (11) while occupying empty buildings as their primary shelter (12). Gang formation and taking part in illegal activities (13) allow for social acceptance in peer groups (14), which is another crucial survival strategy. Street children also engage in different forms of non-violent criminal behavior to survive the street strategy (15). As such, it is worth asking how these children cope on a daily basis.

To deal with the challenges mentioned previously, street children need some personal characteristics that are known to help people deal effectively with adverse situations, such as but not limited to self-esteem and resilience. Exploring self-esteem and resilience among this population with specific reference to differentiating street children from non-street children might shed light on the phenomenon, thus helping researchers, policymakers, and clinicians to formulate intervention strategies to help street children to cope with their adverse situations more adequately, hoping they will eventually leave the streets.

Rosenberg (16) defines self-esteem as a positive or negative attitude a person carries toward themselves. The self-esteem theory indicates that self-esteem serves as a form of a protective factor when an individual is faced with challenging situations (17). As much as self-esteem can be a good or positive feeling about the self, it is influenced by persons in one's life. Early childhood experiences significantly contribute to the development of one's self-esteem (18). Street children have reported poor self-esteem associated with poor subjective well-being (19). In a sample of homeless youth, high self-esteem was found to be related to low levels of psychological distress (20), reducing the risk of mental health problems (21), suggesting that self-esteem serves as a protective factor.

Resilience means the ability to bounce back suggesting that people with high resilience levels are likely to cope better under stressful life situations. Roy et al. (22) reported that resilient children are likely to cope better with adverse life situations as resilience is a protecting factor for them. Meaningfully, Karatas and Cakar (23) argued that when an individual's self-esteem improves, resilience also improves suggesting a link between resilience and self-esteem.

Various factors, including family environment, are associated with the development of resilience among children (24). In South Africa, street children come from unstable abusive family environments (25) which might negatively affect their development of resilience. However, Malindi and Theron (26) and Madu et al. (27) reported that street children are more resilient. Elsewhere, homeless youth have also been found to be resilient (28). They demonstrated this by engaging in peer mutual trust and friendships (29), taking part in street markets (30) finding shelter, and engaging in safer sex (31), thus enabling them to cope with street life.

Research has been conducted among street children populations across the globe, in Africa, and in Limpopo Province in particular (32–35). Most of the available studies focused on pathways from the homes to the streets and challenges during a period of homelessness with a few studies exploring the coping mechanisms. However, in South Africa, despite an increase in the visibility of children on South African streets, this population remains under-investigated. Those who have investigated this situation (34–36), have not explored their differences with children who are not in the streets.

Regardless of the evidence of hardship and trauma among this population (6), studies that explore coping strategies remain limited in South Africa. Few studies (6, 26, 36) explored resilience. More studies that explore resilience and other factors associated with coping strategies among this population are needed to help shed light on appropriate intervention strategies. It is anticipated that this study finding could inform tailor-made intervention strategies to address the challenges faced by this population. It is against this background that the current study explores the difference in self-esteem and resilience among street children, comparing them with children in the general population.

## Materials and Methods

### Participants

A total of 600 (*N* = 600) children, that is 300 street children who resided in the street of the towns of Limpopo Province and 300 children who stayed in their homes, were purposively sampled to take part in this study. The street children sampled comprised 42% males and 58% females with their ages ranging from 8–18 years with the mean age of 15.92 (SD = 1.89). The non-street children sample comprised 56.7 % females and 43.3% males with the age ranging from 8 to 18 years with a mean of 15.46 (SD = 1.87). The street children were compared to the family children.

From the street children population, the youngest to get to the street was at the age of 6 years. The majority (31.7%) of them have been in the streets for more than 2 years, followed by those who have been on the streets for more than 3 years (30.7%). Of the sample, 67.7 % did not have both parents as one (31.0%) or both (37.3%) died. These children are South African (62.7%), Zimbabwean (36.7%), and a few from Mozambique (0.7%) (Table 1). They have at least some high school (36.3%) and some primary school (35.7%) level of education. The reasons for their homelessness were linked to a history of emotional, physical, and sexual abuse while at home.

**Table 1:** Demographic table for street children and non-street children.

	**Street children *N* (%)**	**Non-street children *N* (%)**
**Gender**		
Male	174 (58.0%)	130 (43.3%)
Female	126 (42.6%)	170 (56.7%)
**Age**		
Pre-adolescent	31 (10.3%)	41 (13.7%)
Adolescent	269 (89.7%)	259 (86.3%)
**Level of education**		
Primary school	188 (62.7%)	33 (11.0%)
High school	111 (37.0%)	267 (89.0)
No formal education	1 (0.3%)	
**Nationality**		
South African	188 (67.2%)	300 (100%)
Zimbabwean	110 (36.7%)	
Mozambique	2 (0.7%)	

### Procedure

The data were collected on the streets of various towns in Limpopo Province. This was with the use of a questionnaire that had three sections. Section A contained biographical information, Section B had a self-esteem scale, and Section C had the resilience scale. Participants completed the questionnaires voluntarily with the assistance of the first author and an appointed research assistant who is a registered mental health care professional. The principal investigator and the research assistant helped participants in completing the questionnaires. They read the questions out and clarified some of them, and participants filled in the answer that resonated with them. The participants completed the questionnaire starting from the demographic section to the different measures one after the other as the questionnaire was compiled. They were allowed to rest from completing the questionnaire for about 15–20 min when they needed to do so. They were allowed to continue with the questionnaire after the break.

The study received ethical approval (NWU-00117-10-A3- Mafikeng Health) from the Department of Psychology, Higher Degrees Committee, and Ethics Committee of the North-West University. Additional approval was obtained from the Limpopo Provincial Department of Education. Assent forms were also signed by the participants. For participants who were under the age of 18 years their legal guardians gave consent where applicable, and those participants on the streets only gave assent for participation.

### Materials

#### Rosenberg Self-Esteem Scale

This 10-item four-point Likert scale assessed the children's self-esteem (16). The items are scored differently with items 1, 2, 4, 6, and 7 scored from 0, which means strongly disagree, to three, which means strongly agree. Item 3, 5, 8, 9, and 10 are reverse scored with 0 indicating strongly agree and three indicating strongly disagree. The scores range from 0 to 30 with 30 being the possible highest score suggesting high self-esteem. The sample items include “on the whole, I am satisfied with myself” and “at times I think I'm no good at all.” This scale has been widely used across many populations of different demography. It is reliable 0.78 and 0.92 in South Africa (37) and also in the current study with an alpha of 0.65.

#### Connor–Davidson Resilience Scale

This 25-item scale was used to measure resilience (38). Each item is rated on five-point frequency response ranging from 0 (“not true at all”) to 4 (“true nearly all the time”). The total score is between 0 and 100 with a score of 51 and above suggesting greater resilience. Singh and Yu (39) found the overall alpha reliability of α = 0.89, whereas in the current study the Cronbach's' alpha reliability was 0.90.

### Data Analysis

The statistical analysis was carried out using the Statistical Package for Social Science (SPSS 22). With this software, data were computed using an independent sample *t*-test to test the hypothesis about the difference in self-esteem and resilience between the two groups. Chi-square was also computed to determine gender and gender differences in self-esteem and resilience between the two groups.

## Results

### Self-Esteem and Resilience Differences

The results of the independent sample *t*-test revealed significant statistical differences in self-esteem [*t*_(598)_ = 20.03, *p* < 0.000] between the two groups of street children and family children as indicated in Table 1. Further analysis of mean scores indicates that street children have poor self-esteem (mean = 13.44, SD = 4.06) compared to family children (mean = 19.77, SD = 3.67).

Furthermore, an independent sample *t*-test statistical analysis method was used to test the differences in resilience between these two groups. The results showed significant statistical differences in resilience [*t*_(598)_ = 9.48, *p* < 0.000] between these two samples (see Table 2). Additionally, analysis of mean scores indicates that street children reported poor resilience (mean = 56.03, SD = 15.54) compared to family children who scored higher on resilience (mean = 67.50, SD = 14.06).

**Table 2:** Independent sample *t*-test analysis on self-esteem and resilience.

	**Street children**		**Control group**			
**Variable**	**Mean**	**SD**	**Mean**	**SD**	***T***	***P***
SE	13.44	4.058	19.77	3.667	20.036	0.000^***^
*R*	56.03	15.542	67.50	14.075	9.480	0.000^***^

*SE, self-esteem; R, resilience*.

*** *p < 0.000*.

A chi-square analysis was computed to determine gender and age difference in self-esteem and resilience between the two groups (see Table 3 below). Male $[X_{\left(\text{1},N\text{=174}\right)}^{2}=$ = 94.82, *p* = 0.000] and female $[X_{\left(\text{1},N\text{=126}\right)}^{2}=$ = 93.40, *p* = 0.000] street children reported poor self-esteem compared to their non-street children counterparts. The same gender differences were further observed on resilience scores as male $[X_{\left(\text{1},N\text{=174}\right)}^{2}=$ = 39.64, *p* = 0.000] and female $[X_{\left(\text{1},N\text{=126}\right)}^{2}=$ = 23.16, *p* = 0.000] street children also reported poor resilience compared to the control.

**Table 3:** Results showing gender and age differences in self-esteem and resilience between the street children and non-street children.

	**Total *N* (Mean; SD)**	**Street children *N* (Mean; SD)**	**Non-street children *N* (Mean; SD)**	**X**^**2**^	***P***
**Self-esteem**					
**Gender**					
Male	304 (1.54; 0.49)	174 (1.30; 0.45)	130 (1.86; 0.34)	94.82	0.000^***^
Female	296 (1.60; 0.49)	126 (1.29; 0.45)	170 (1.84; 0.36)	93.40	0.000^***^
**Age**					
Pre-adolescent	72 (1.58; 0.49)	31 (1.32; 0.47)	41 (1.78; 0.41)	94.82	0.000^***^
Adolescent	528 (1.57; 0.49)	269 (1.29; 0.45)	259 (1.88; 0.45)	93.40	0.000^***^
**Resilience**					
**Gender**					
Male	304 (1.71; 0.45)	174 (1.57; 0.497)	130 (1.90; 0.30)	39.64	0.000^***^
Female	296 (1.80; 0.40)	126 (1.67; 0.473)	170 (1.89; 0.30)	23.16	0.000^***^
**Age**					
Pre-adolescent	72 (1.74; 0.44)	31 (1.68; 0.475)	41 (1.78; 0.41)	0.965	0.237 (NS)
Adolescent	528 (1.76; 0.43)	269 (1.60; 0.490)	259 (1.92; 0.27)	60.94	0.000^***^

*** *p < 0.000*.

Both pre-adolescent $[X_{\left(\text{1},N\text{=31}\right)}^{2}=$ = 94.82, *p* = 0.000] and adolescent $[X_{\left(\text{1},N\text{=269}\right)}^{2}=$ = 93.40, *p* = 0.000] street children reported poor self-esteem when compared to their non-street children age mates. The street children in the adolescent group $[X_{\left(\text{1},N\text{=269}\right)}^{2}=$ = 60.94, *p* = 0.000] reported poor resilience where are there were no statistical difference in the pre-adolescent group when resilience was assessed.

## Discussion

The main study aim was to explore self-esteem and resilience differences between street children and non-street children in the general population. An independent group sample design was chosen to explore group differences. The results revealed that street children reported low self-esteem compared to their non-street children counterparts suggesting challenges associated with self-esteem. These results are supported by Maccio and Schuler (40), who stated lower levels of self-esteem among the homeless youth compared to their non-homeless counterparts in the USA. Contrary to this finding Ezeokana et al. (41) in Nigeria reported no difference in self-esteem between street children and non-street children. Further contradictions were reported by Tozer et al. (42) as street youth had a good sense of self-worth demonstrated by their ability to refrain from substance abuse longing for a better future. The reasons these discrepancies in literature for the self-esteem and resilience could be attributed to the different types of street children that exists as Madu et al. (27) indicated that there are hard-core, sheltered, and part-time street children. Issues related to social stigma which are common among this population (43) also contribute. Those less stigmatized usually benefit from social support (44) by means of maintaining contact with their families. This suggests that future studies should explore different types of street children and their dynamics. Different street children reported different history of adversity (45) which could impact on their self-esteem and resilience.

Despite this discrepancy in literature, the current study finding suggests that the street children population remains at risk due to their low self-esteem suggesting the reason for prolonged periods of homelessness. To curb this challenge, street children need to be helped with self-esteem as it serves as a protective factor for those who experienced adversities (44, 46).

The self-esteem theory which indicates that self-esteem is a result of positive appraisal by significant others (47) can help with a better understanding of the current results. In this case, due to common reasons for homelessness, such as poverty, neglect, family breakdown, death of one or both parents, and other forms of child abuse (8, 48, 49), these children lack that positive appraisal. This could also be attributed to the fact that a majority of the samples had no parents who usually serve as a foundation of self-esteem. This is seen in other studies, where street children have been reported to have been abused by their parents who were supposed to protect them and enhance their self-esteem (50, 51). Furthermore, in most instances, street children have no family ties and spend most of the time of their lives worrying about their survival and not about their worth.

This study revealed that street children reported poor resilience compared to non-street children. These results concur with the work of Cleverley and Kidd (52) who found lower resilience among homeless youth. Regardless of these findings, there is still evidence of large numbers of street children in many South African cities (26). This could be associated with their resilience which was reported by Malindi (36). This indicates that, as much as street children in this current study reported poor resilience compared to their non-street children counterparts, this does not mean that they are not resilient. They are found not to be resilient only when a comparison with their non-street children counterpart is made. This is supported by other studies that have revealed that, even when some street children reported psychological problems such as post traumatic stress disorder, they were still found to be resilient (53). Despite evidence of harsh living conditions, street children have shown adaptability as the majority of them have lived on the streets for years in the current study, up to 7 years.

Evidence that somehow street children can cope with their living conditions may help answer this question. They use personal character and emotional strength, cultural values, religious beliefs, and peer support (6). This is also in line with their responses on the resilience scale as some answered positively to questions about seeking help from friends and praying about their situation. They support each other during difficult times, and that helps them survive harsh street life (54). Furthermore, due to the available peer support these children offer to each other and other forms of support received from people who provide them with money and food (55, 56) or other family and community capacities (57), they can be regarded as resilient as such relationships promoter resilience (58).

It is worthy to note that the street children in this sample continued to report low scores on self-esteem and resilience when compared to their non-street children counterparts in terms of gender and age. Male and female adolescent street children reported poor self-esteem and resilience when compared to non-street children. The pre-adolescent street children reported poor self-esteem when compared to non-street children, but no significant statistical difference was found between pre-adolescent street children and non-street children with resilience. These results stress the different levels of self-esteem and resilience between these two groups. A possible explanation for these findings could be the continued adversity faced by this group while still at home (59) and while on the streets (60). This suggests that treatment intervention plans should be inclusive of all street children regardless of age and gender.

### Limitations

The cross-sectional design and nature of this study serve as a limitation. Data were collected once-off at a single point in time minimizing the possibilities of establishing causalities. The children solely reported on the data self-report, and data from parents or guardians could not be collected. There also might have been a poor recall of childhood memories leading to inaccurate reporting. Other extraneous variables such as temperament and coping styles were not explored.

Several strengths have been noted in this study. Data were collected from those children who were on the streets surviving mainly on their own rather than from those children who are in care centers who receive help in different forms. It is argued that their survival and coping strategies are thus not influenced by the help they receive in care centers. South Africa has an estimate of 250,000 street children (61) with an increase of 0.002 to 0.22 in Limpopo Province (62). This makes the sample size of the study big enough to generalize the findings for the Limpopo Province which could be extended to other provinces.

## Conclusions

This study concludes that there is a significant difference between street children and non-street children. Street children differed from non-street children in terms of self-esteem and resilience where street children reported low self-esteem and poor resilience. However, this does not take away from the fact that street children demonstrated to some extent a level of self-esteem and resilience even though it was not statistically significant.

## Suggestions for Improvements

Future studies should consider exploring other extraneous variables such as socio-demographic factors that could influence the street children's survival method. History of substance use or use should also be considered. Longitudinal studies can provide a clearer pattern on self-esteem and resilience of this population helping in the formation of intervention strategies over time.

## Data Availability Statement

The datasets generated for this study are available on request from the corresponding author.

## Ethics Statement

The studies involving human participants were reviewed and approved by North-West University Ethics Committee. Written informed consent from the participants' legal guardian/next of kin was not required to participate in this study in accordance with the national legislation and the institutional requirements.

## Author Contributions

MM has developed the study concept, study design, sought ethical clearance, data collection, capturing and analysis were performed, wrote this manuscript, contributed to the article and approved the submitted version.

## Conflict of Interest

The author declares that the research was conducted in the absence of any commercial or financial relationships that could be construed as a potential conflict of interest.
